# Nalmefene, an opioid receptor modulator, aggravates atherosclerotic plaque formation in apolipoprotein E knockout mice by enhancing oxidized low-density lipoprotein uptake in macrophages

**DOI:** 10.1016/j.bbrep.2024.101688

**Published:** 2024-03-23

**Authors:** Mitsuhisa Koga, Koshun Inada, Ayano Yamada, Kana Maruoka, Atsushi Yamauchi

**Affiliations:** a Department of Drug Delivery, Faculty of Pharmaceutical Sciences, Fukuoka University, 8-19-1 Nanakuma, Jonan-ku, Fukuoka, 814-0180, Japan; b Department of Pharmaceutical Care and Health Sciences, Faculty of Pharmaceutical Sciences, Fukuoka University, 8-19-1 Nanakuma, Jonan-ku, Fukuoka, 814-0180, Japan

**Keywords:** Nalmefene, Atherosclerosis, Side effect, Opioid receptors

## Abstract

Nalmefene, an antagonist of mu- and delta-opioid receptors and a partial agonist of kappa-opioid receptors, has shown promise in reducing alcohol consumption among patients with alcohol dependence. Opioid receptors play pivotal roles in various physiological processes, including those related to peripheral inflammatory diseases such as colitis and arthritis, as well as functions in the immune system and phagocytosis. Atherosclerosis, a chronic inflammatory disease, progresses through the phagocytosis and uptake of oxidized low-density lipoprotein (oxLDL) by macrophages in atherosclerotic plaques. Despite this knowledge, it remains unclear whether nalmefene influences the formation of atherosclerotic plaques and increases the risk of serious cardiovascular events. This study aims to elucidate the impact of nalmefene on atherosclerosis in apolipoprotein E knockout (ApoE KO) mice and peritoneal macrophages in vitro.

In this experiment, 8-week-old male ApoE KO mice were fed a high-fat diet intraperitoneally administered either vehicle (saline) or nalmefene (1 mg and 3 mg kg^−1^ day^−1^) for 21 days. Oil red O-staining and immunohistochemistry with an anti-MOMA2 (monocyte/macrophage) antibody showed that a dose-dependent increase in atherosclerotic plaque formation and augmentation of macrophage-rich plaque formation in ApoE-KO mice. Further investigations focused on the effects of nalmefene on the expression of scavenger receptor CD36 in RAW264.7 cells, conducted through western blotting analysis. Nalmefene demonstrated a significant increase in CD36 protein expression in RAW264.7 cells. To explore the impact on oxidized LDL uptake in peritoneal macrophages, cells were treated with nalmefene (300 μg/mL) for 24 h, followed by the addition of DiI-labeled oxLDL (DiI-oxLDL) for 4 h. Nalmefene significantly enhanced DiI-oxLDL uptake in macrophages. Additionally, treatment with nalmefene (300 μg/mL) for 24 h decreased the mRNA expression of mu-, delta-, and kappa-opioid receptors in RAW264.7 cells.

In conclusion, nalmefene may augment oxLDL uptake by macrophages through increased CD36 expression and decreased opioid receptor, thereby contributing to atherosclerotic plaque formation and vulnerability. Consequently, the use of nalmefene may be associated with an elevated risk of cardiovascular events.

## Highlights

• Nalmefene raises total cholesterol and triglyceride levels in ApoE KO mice.
• Nalmefene promotes atherosclerotic and macrophage-rich plaques in ApoE KO mice.
• Nalmefene boosts oxLDL uptake by macrophages through increased CD36 expression.
• Nalmefene may elevate the risk of cardiovascular events, including atherosclerosis.

## 1 Introduction

Chronic heavy alcohol consumption is associated with various diseases and complications, including cardiovascular diseases, liver diseases, and cancer [[1], [2], [3]]. Cessation of alcohol consumption is crucial to preventing the onset and progression of cardiovascular diseases. However, it remains challenging for patients with alcohol dependence to quit drinking. Therefore, the use of nalmefene, a drug that reduces alcohol consumption in individuals with alcohol dependence, is recommended as a treatment [4,5]. Nalmefene acts as an antagonist of mu- and delta-opioid receptors and a partial agonist of kappa-opioid receptors [6]. It has been shown to significantly reduce monthly total alcohol consumption and the number of heavy drinking days [7,8].

Atherosclerotic plaque formation is a key factor in the development of cardiovascular disease. Oxidized low-density lipoprotein (oxLDL) contributes to the formation and progression of atherosclerotic plaques by accumulating lipids in macrophages. Macrophages, in turn, form cells, and the uptake of oxLDL in macrophages within atherosclerotic plaques can lead to plaque rupture and vulnerability. Scavenger receptors such as CD36, lectin-like oxLDL receptor-1 (LOX-1), and scavenger receptor class A (SR-A) play critical roles in the uptake of oxidized low-density lipoprotein (oxLDL) by macrophages [9,10].

Opioids exert various effects on immune cells, such as enhancing cytotoxicity and stimulating monocyte chemotaxis [11]. Delta opioid receptor agonists directly act on immune cells to mitigate colonic inflammation [12]. The endogenous opioid peptide Met-enkephalin inhibits phagocytosis by mouse peripheral macrophages through the delta-opioid receptor [13]. Additionally, morphine, an opioid receptor agonist, inhibits phagocytosis in peritoneal macrophages of mice [14] and elevates plasma levels of total cholesterol and LDL cholesterol in rats on a cholesterol-cholic acid-supplemented diet [15]. Naltrexone, a mu-opioid receptor antagonist, prevents morphine-induced hyperlipidemia [16]. Hence, opioids and opioid receptors play roles in peripheral inflammation, the immune system, phagocytosis, and the modulation of plasma cholesterol levels.

However, it remains unclear whether nalmefene, an opioid receptor modulator, is associated with the development of atherosclerosis and an increased risk of serious cardiovascular events. In this study, to assess whether nalmefene treatment affects cardiovascular events, especially atherosclerotic plaque formation, we investigated the impact of nalmefene on atherosclerotic plaque development in apolipoprotein E knockout (ApoE KO) mice and macrophages in vitro.

## 2 Material and methods

### 2.1 Animals and experimental design

Male ApoE KO mice on C57BL/6J background were obtained from Jackson Laboratory (Bar Harbor, ME, USA) and provided food and water ad libitum. All animals were housed in a specific pathogen-free and 12:12 h light-dark cycle environment. The study protocol (No. 2009043) was approved by the Laboratory Animal Care and Use Committee of Fukuoka University.

Eight-week-old ApoE KO mice were fed high-fat diet containing 1.25% cholesterol, 15% cacao butter, and 0.5% sodium cholate (Oriental Yeast Co., Tokyo, Japan). The mice were randomized to saline (vehicle)- or nalmefene-treated mice. Nalmefene (Focus Biomolecules, Plymouth Meeting, PA, USA) was administered subcutaneously at doses of 1 or 3 mg kg^−1^ day^−1^ for every day. After 3-week treatment, the mice were euthanized under anesthesia, and their aortas were collected to assess the effect of nalmefene on atherosclerotic plaques.

### 2.2 Measurement of plasma levels of total cholesterol and triglyceride

At 11 weeks of age, blood was collected to measure the plasma levels of total cholesterol and triglycerides using LabAssay™ (Fujifilm Wako Pure Chemical Corporation, Osaka, Japan).

### 2.3 En-face plaque area

Immediately after mice were sacrificed, the aorta was excised for quantification of en-face plaque area [17,18]. Briefly, we carefully removed fat and adventitious tissue, longitudinally opened the entire aorta, pinned it with a black wax surface, and stained it with Oil Red O (Sigma, St. Louis, MO, USA). En-face images were obtained using a digital camera and analyzed using the public domain software ImageJ (NIH Image, Bethesda, MD, USA) to quantify the percentage of luminal surface plaques.

### 2.4 Histology and immunohistostaining

Hearts collected after ice-cold PBS perfusion were fixed in 4% paraformaldehyde phosphate buffer solution at 4 °C overnight and embedded in O.C.T. Compound (Sakura FineTech, Tokyo, Japan). Serial frozen sections (7-μm thickness) of the aortic root were prepared as previously described [17,18]. Atherosclerotic plaques were investigated in five sets of sections, with each set separated by 70 μm. The sections were stained oil Red O to identify the lipid-rich core. Immunohistochemical staining utilized a rat anti-monocyte/macrophage (MOMA2) antibody (1:300, BMA Biomedicals, Augst, Switzerland) and a Vectastain Elite ABC HRP kit (Vector Laboratories, Burlingame, CA, USA). The bound antibody was visualized by the indirect immunoperoxidase method using 3-amino-9-ethylcarbazole (AEC; Vector Laboratories). Aortic root images were captured using a Keyence BZ-X710 microscope (Keyence Corporation, Osaka, Japan). Oil Red O- and MOMA2-positive areas were analyzed using ImageJ software. For each mouse, the mean values from five independent sections were employed for the analysis.

### 2.5 Cell culture

The murine macrophage line RAW 264.7 (Riken, Saitama, Japan) was seeded in 60-mm dishes at 8 × 10^5^ cells/dish. Cells were cultured in a complete medium (RPMI 1640 medium containing 10% fetal bovine serum and 100 U/mL penicillin/streptomycin) in a humidified atmosphere of 5% CO_2_/95% air at 37 °C. RAW264.7 cells were serum-starved for 3 h and treated with nalmefene (0–300 μg/mL) for 24 h.

### 2.6 Western blot analyses

Proteins of the mice aorta or RAW264.7 cells were isolated using lysis buffer containing 50 mM HEPES, 50 mM sodium chloride, 5 mM ethylenediaminetetraacetic acid, 1% Triton X-100, 1 mM sodium orthovanadate, 50 mM sodium fluoride, 10 mM sodium pyrophosphate decahydrate, and 1 mM phenylmethylsulfonyl fluoride, and 1% protease inhibitor cocktail (Nacalai Tesque, Kyoto, Japan). After centrifugation at 12,000×*g* for 15 min at 4 °C, the supernatants were collected and as total proteins in aorta and cells. The protein concentrations were measured with a BCA assay kit (Thermo Fisher scientific, Waltham, MA, USA). Equal amounts of total protein (20 μg/sample) were separated by sodium dodecyl sulfate–polyacrylamide gel electrophoresis (10.5%) and electro-transferred to polyvinylidene difluoride membranes (Millipore, Billerica, MA, USA). The membrane was blocked with Blocking One (Nacalai Tesque) for 60 min at room temperature. The blot was incubated with goat anti-CD36 (1:500; R&D Systems, Minneapolis, MN, USA) and mouse anti-glyceraldehyde 3-phosphate dehydrogenase (GAPDH; 1:2000; Millipore) antibodies at 4 °C overnight, and then incubated with horseradish peroxidase-conjugated anti-goat and -mouse (1:10000 dilution, Bio-Rad Laboratories, Hercules, CA, USA) antibodies for 60 min at room temperature. Proteins were detected by ImmunoStar® LD (Fujifilm Wako Pure Chemical Corporation). Signal intensities were normalized using GAPDH. Band Images were digitally captured with a FluorChem SP imaging system (Alpha Innotech, San Leandro, CA, USA) and bands intensities were quantified using ImageJ software.

### 2.7 OxLDL uptake in mouse peritoneal macrophages

Mouse peritoneal macrophages from female C57BL/6 mice were elicited by intraperitoneal injection of 2 mL of 4% thioglycollate medium (Sigma) 3 days before collecting. The cells were incubated in 24-well plates at 5 × 10^5^ cells/well in RPMI 1640 complete medium in a humidified atmosphere of 5% CO_2_/95% air at 37 °C. After 3 h, non-adherent cells were washed away three times with PBS. The adhered peritoneal macrophages were incubated in medium for 24 h before use. To evaluate the effect of nalmefene on oxLDL uptake, peritoneal macrophages were treated with nalmefene (300 μg/mL) for 24 h after serum starvation for 3 h and then 5 μg/mL DiI-labeled oxLDL (Thermo Fisher Scientific) was added to the culture medium for 4 h.

After three washes with PBS, the cells were fixed in 4% paraformaldehyde phosphate buffer solution and mounted with Vectashield containing the nuclear dye DAPI (Vector Laboratories). The area of DiI-labeled peritoneal macrophages was measured, and the number of peritoneal cells was counted. OxLDL uptake by peritoneal macrophages was evaluated by the DiI-positive area per cell in 15 random microscopic fields from three independent wells in each experiment. The experiment was repeated seven times, and the mean areas from separate experiments were analyzed.

### 2.8 Real-time quantitative PCR

The total RNA was extracted from RAW264.7 cells treated with nalmefene (300 μg/mL) for 24 h using the RNeasy Mini RNA Extraction Kit (Qiagen, Valencia, CA, USA) following the manufacturer's instructions. Subsequently, 1 μg of RNA was reverse-transcribed with the Superscript VILO Synthesis Kit (Invitrogen, Carlsbad, CA, USA) using random primers. The cDNA was subjected to real-time qPCR on an MX3000P real-time PCR system (Agilent Technologies, Santa Clara, CA, USA) and normalized to the housekeeping gene, GAPDH. The following primers were used for PCR reactions (forward and reverse, respectively): mu-opioid receptor (5′-CGA ACA CTC TTG AGT GCT CTC A-3′and 5′-GCT GTC CAT GGT TCT GAA TGC TT-3′), delta-opioid receptor (5′-GGA AGC AGA GCT GGT GAT TCC T-3′ and 5′-TCC TGG TTC CTG GAG CTG GAA T-3′), kappa-opioid receptor (5′-CTT CCA GTC TTG GAA GGC ACA A-3′ and 5′-CAA GTC ACC GTC AGC TTT CCA A-3′), and GAPDH (5′-AAA GAC CCC TTC ATT GAC-3′ and 5′-TCC ACG ACA TAC TCA GCA C-3′).

### 2.9 Statistical analyses

The results are presented as mean ± standard deviation (SD). Differences between the two groups were assessed using the unpaired Student's *t*-test. Statistical comparisons among multiple groups were conducted using ANOVA followed by Tukey's multiple comparison test. A significance level of *P* < 0.05 was considered statistically significant.

## 3 Results

### 3.1 Plasma levels of total cholesterol and triglyceride in ApoE KO mice

We investigated the effect of nalmefene, administered at doses of 1 mg and 3 mg kg^−1^ day^−1^ for 21 days, on the plasma levels of total cholesterol and triglycerides in ApoE KO mice.

Plasma levels of total cholesterol for vehicle, 1 mg, and 3 mg kg^−1^ day^−1^ nalmefene were 1187.95 ± 266.18 mg/dL, 1362.28 ± 262.91 mg/dL, and 1537.17 ± 420.61 mg/dL, respectively (Table 1). Nalmefene at a dose of 3 mg kg-1 day^−1^ significantly elevated total cholesterol compared to the vehicle treatment (*P* < 0.05). Additionally, plasma levels of triglycerides for vehicle, 1 mg, and 3 mg kg^−1^ day^−1^ nalmefene were 298.12 ± 100.57, 420.10 ± 129.03, and 435.05 ± 151.12 mg/dL, respectively (Table 1). Nalmefene at doses of 1 mg and 3 mg kg^−1^ day^−1^ significantly increased triglyceride levels compared to the vehicle treatment (each *P* < 0.05).

**Table 1:** Plasma levels of total cholesterol and triglyceride in ApoE KO mice. Data are expressed as the mean ± SD (n = 8–9). *P < 0.05 vs vehicle.

	vehicle	Nalmefene 1 mg kg^−1^ day^−1^	Nalmefene 3 mg kg^−1^ day^−1^
Total Cholesterol (mg/dL)	1187.95 ± 266.18	1362.28 ± 262.91	1537.17 ± 420.61*
Triglyceride (mg/dL)	298.12 ± 100.57	420.10 ± 129.03*	435.05 ± 151.12*

### 3.2 Effect of nalmefene on atherosclerotic plaque formation in ApoE KO mice

We investigated the potential of nalmefene to induce atherosclerotic plaque formation in ApoE-KO mice. After administering nalmefene at doses of 1 mg and 3 mg kg^−1^ day^−1^ for 21 days, we observed a dose-dependent progression of atherosclerotic plaque formation in the whole aorta and aortic arch of ApoE KO mice.

**Fig. 1:**
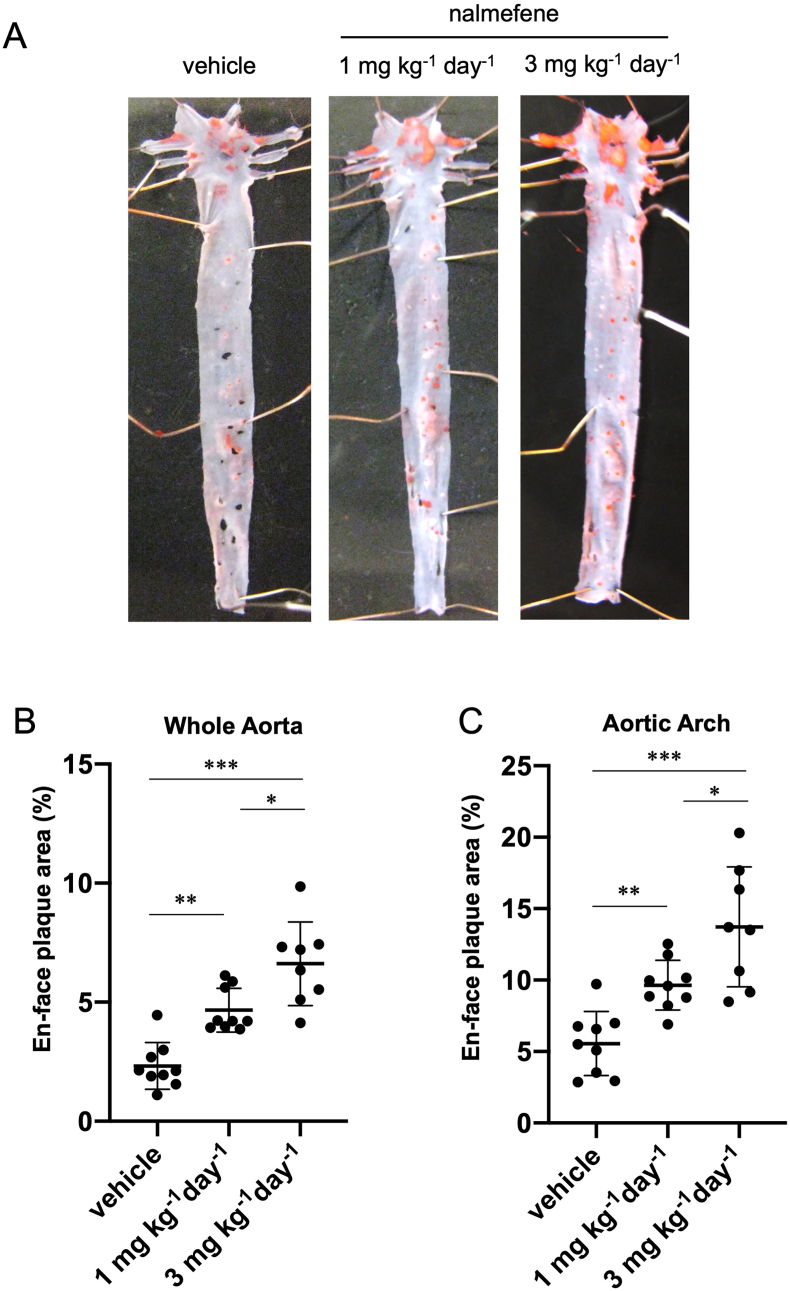
Atherosclerotic plaque formation in the aorta of nalmefene-treated apolipoprotein E knockout mice. Representative en face photographs of the aorta showing oil red O-stained atherosclerotic lesions (red) (A). Quantitative analysis of the Oil Red O-positive area in the whole aorta (B) and aortic arch (C). Each dot shows data obtained from each mouse. Data represent the means ± SD (n = 8–9). *P < 0.05, **P < 0.01, ***P < 0.001. (For interpretation of the references to color in this figure legend, the reader is referred to the Web version of this article.)

The en-face plaque area in the whole aorta increased approximately 2.2-fold and 3.4-fold after nalmefene treatment at 1 mg and 3 mg kg^−1^ day^−1^, respectively, compared to the vehicle (*P* < 0.05 and *P* < 0.01, respectively; Fig. 1B). Additionally, the en-face plaque area in the aortic arch increased approximately 1.7-fold and 2.5-fold after nalmefene treatment at 1 mg and 3 mg kg^−1^ day^−1^, respectively, compared to the vehicle (*P* < 0.05 and *P* < 0.01, respectively; Fig. 1C).

**Fig. 2:**
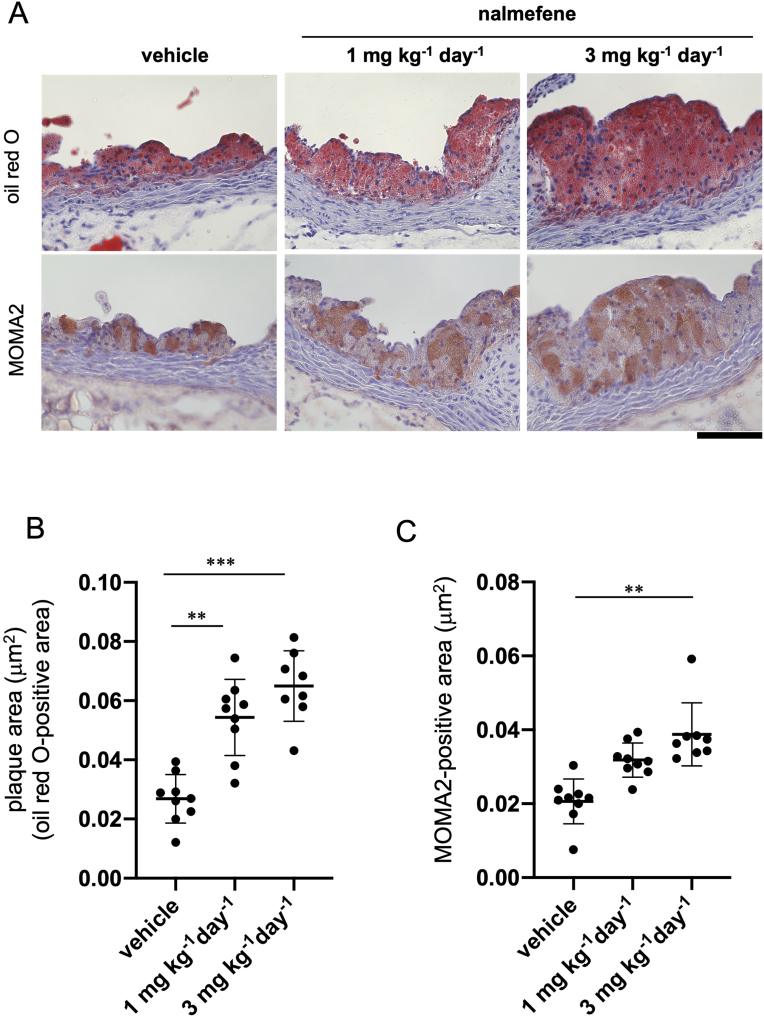
Atherosclerotic plaque formation in the aortic root of apolipoprotein E knockout mice treated with vehicle and nalmefene (1 mg or 3 mg kg^−1^ day^−1^) for 21 days. (A) Representative photomicrographs of oil red O (red color in the upper panels) and MOMA2 (monocyte/macrophage)-stained (brownish red color in the lower panels). Bar; 100 μm. (B) The oil red O-positive and (C) the MOMA2-positive areas (mm^2^) in the aortic root were measured in 5 separate sections (7 μm). Each dot shows data obtained from each mouse. Data represent means ± SD (n = 8–9). **P < 0.01, ***P < 0.001. (For interpretation of the references to color in this figure legend, the reader is referred to the Web version of this article.)

Furthermore, after administering nalmefene at doses of 1 mg or 3 mg kg^−1^ day^−1^ for 21 days, there was a notable increase in atherosclerotic plaque formation in the aortic root of ApoE KO mice when compared to the control (Fig. 2A and B) (*P* < 0.05 and *P* < 0.01, respectively). Additionally, the MOMA2-positive area in the aortic root of ApoE KO mice significantly increased with the higher dose (3 mg kg^−1^ day^−1^) but not with the lower dose (1 mg kg^−1^ day^−1^) when compared to that in the control (Fig. 2A and C) (*P* < 0.01).

### 3.3 Effect of nalmefene on the expression of scavenger receptors in RAW264.7 cells

To investigate the mechanism underlying the augmentation of macrophage-rich plaque formation by nalmefene, we assessed the expression of scavenger receptors, including CD36, in nalmefene-treated RAW264.7 cells through western blot analysis.

**Fig. 3:**
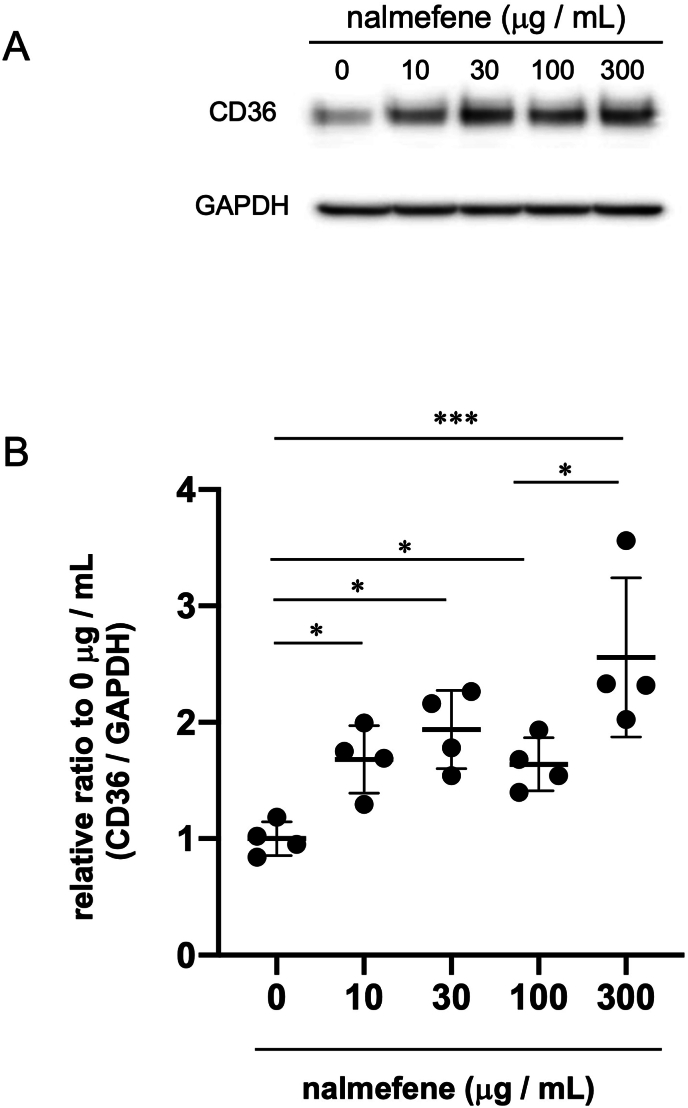
Effect of nalmefene on the protein expression levels of CD36 in RAW264.7 cells. Cells were treated with nalmefene (0–300 μg/mL) for 24 h. (A) Representative immunoblots showing the protein expression levels of CD36. (B) Quantitative analysis of CD36 expression GAPDH was used as a loading control. Each dot shows data obtained from individual experiment. Data represent means ± SD (n = 4) *P < 0.05, ***P < 0.001.

The expression of CD36 protein in RAW264.7 cells exhibited a dose-dependent increase with nalmefene, reaching its peak at 300 μg/mL (Fig. 3). Consequently, we selected 300 μg/mL for subsequent experiments.

### 3.4 Effect of nalmefene on oxLDL uptake in peritoneal macrophages

**Fig. 4:**
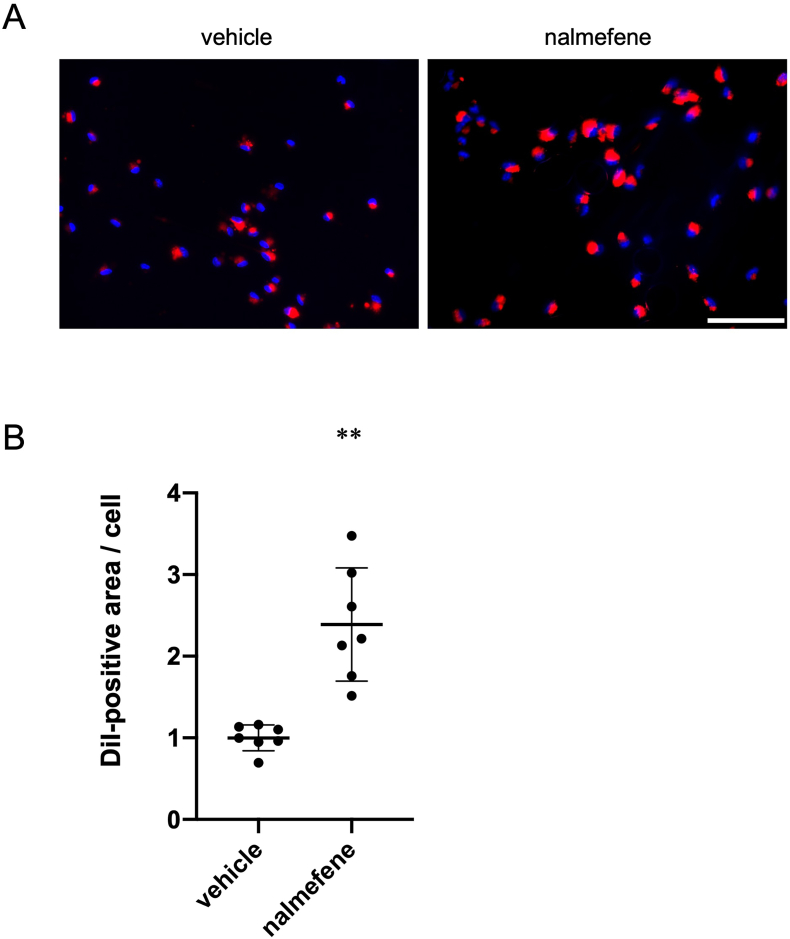
Effect of nalmefene on oxLDL uptake in peritoneal macrophages. Peritoneal macrophages were treated with nalmefene (300 μg/mL) for 24 h and then 5 μg/mL DiI-labeled oxLDL was added to the culture medium for 4 h. (A) Representative images showing incorporated oxLDL in macrophages (red). Bar; 100 μm. (B) Quantitative analysis of DiI-positive and oxLDL-incorporated areas. The results are expressed as the DiI-labeled area per cell. Each dot represents data obtained from individual experiments. Data represent means ± SD (n = 7). **P < 0.01. (For interpretation of the references to color in this figure legend, the reader is referred to the Web version of this article.)

We proceeded to examine the influence of nalmefene on oxLDL uptake by macrophages, given that nalmefene upregulates CD36 expression, which is crucial for incorporating oxLDL into macrophages and contributing to atherosclerosis development. As depicted in Fig. 4, a 24-h treatment with nalmefene (300 μg/mL) resulted in a significant 2.1-fold increase in the DiI-positive area in macrophages compared to vehicle treatment (*P* < 0.05). Thus, nalmefene enhances oxLDL uptake by macrophages and promotes phagocytosis.

### 3.5 Effect of nalmefene on expression of opioid receptors in RAW264.7 cells

**Fig. 5:**
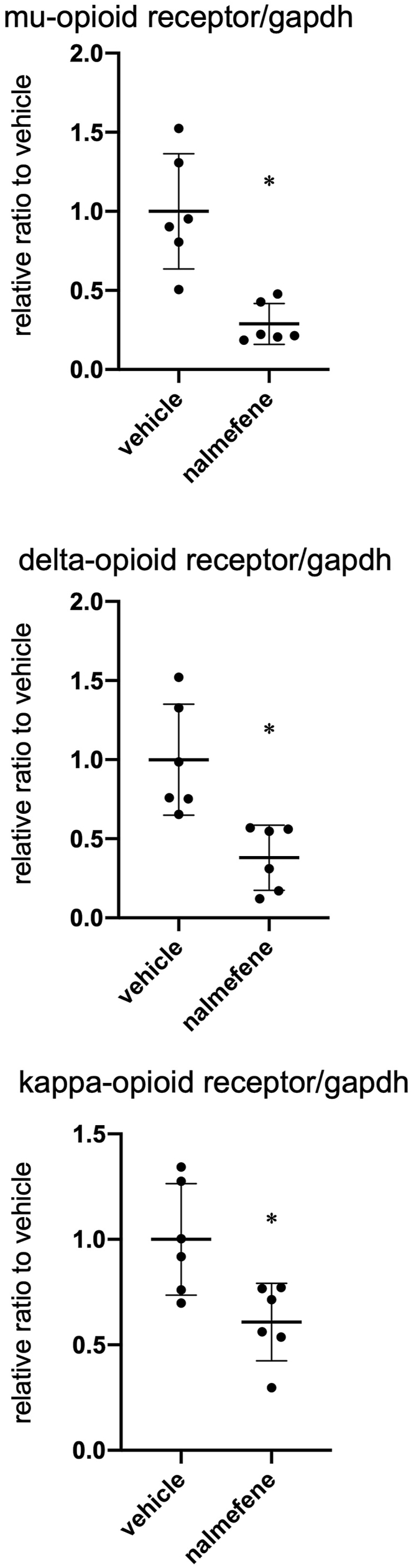
Effect of nalmefene on mRNA expression of opioid receptors in RAW264.7 cells. Cells were treated with nalmefene (300 μg/mL) for 24 h. Dot graph shows quantitative real-time RT-PCR analyses of mRNA expression of mu- (A), delta- (B), and kappa-opioid receptors (C). Data represent means ± SD (n = 6). *P < 0.05.

We also assessed the impact of nalmefene on the expression of mu-, delta-, and kappa-opioid receptors in RAW264.7 cells. After treating with nalmefene (300 μg/mL) for 24 h, there was a significant decrease in the mRNA levels of mu-, delta-, and kappa-opioid receptors by 73%, 56%, and 39%, respectively (Fig. 5 each *P* < 0.05).

## 4 Discussion

Opioids and opioid receptors play roles in peripheral inflammation, immunity, and phagocytosis [11], with these effects being associated with the development of atherosclerotic plaques. Despite this association, it remains unknown whether nalmefene, an antagonist of mu- and delta-opioid receptors and a partial agonist of the kappa-opioid receptor, contributes to the development of atherosclerosis and an increased risk of serious cardiovascular events. In this study, we aimed to determine if nalmefene elevates the risk of cardiovascular events using ApoE KO mice and subsequently elucidate the underlying mechanisms. Our findings suggest that nalmefene promotes the formation of atherosclerotic plaques, thereby increasing the risk of cardiovascular events.

Nalmefene, administered at doses of 1 and 3 mg kg^−1^ day^−1^ for 21 days, exacerbated atherosclerotic plaque formation (Fig. 1, Fig. 2) and elevated plasma levels of total cholesterol and triglycerides (Table 1) in ApoE KO mice. The observed increase in atherosclerotic plaque formation induced by nalmefene may be attributed to the concurrent rise in plasma levels of total cholesterol and triglycerides. This association is plausible, as elevated plasma cholesterol is known to contribute to atherosclerotic plaque development. In contrast, morphine, an opioid agonist, has been reported to increase plasma levels of total cholesterol and LDL cholesterol in rats fed a cholesterol-cholic acid-supplemented diet by reducing bile acid levels in the bile [15]. Consequently, nalmefene might influence bile acid levels, thereby promoting atherosclerotic plaque formation. Notably, naltrexone, a mu-opioid receptor antagonist, has demonstrated the ability to prevent morphine-induced hyperlipidemia [16]. The specific interplay among mu-, delta-, and kappa-opioid receptors is crucial, considering that naltrexone targets the mu-opioid receptor, while nalmefene acts as an antagonist for mu- and delta-opioid receptors and a partial agonist for the kappa-opioid receptor. Despite these insights, the mechanism through which nalmefene increases plasma levels of cholesterol and triglycerides, as well as the involvement of specific opioid receptors, remains unclear. Further experiments are essential to elucidate the precise mechanism of action.

The formation of foam cells from macrophages is recognized as a pivotal process in early atherosclerosis. Macrophages incorporate oxLDL into cells through scavenger receptors, including CD36, LOX-1, and SR-A, ultimately leading to foam cell development [9,10]. In our investigation, we focused on the expression of the CD36 scavenger receptor and oxLDL uptake in macrophages in vitro, as nalmefene was found to increase the formation of macrophage-rich plaques in the aortic roots of ApoE KO mice (Fig. 2). Results showed that nalmefene dose-dependently increased the expression of CD36 (Fig. 3) and enhanced oxLDL uptake by macrophages in vitro (Fig. 4). Consequently, nalmefene may directly contribute to the formation and destabilization of atherosclerotic plaques by promoting oxLDL uptake through increased CD36 expression by macrophages. In contrast, the expression of the other scavenger receptors, LOX-1 and SR-A, in RAW264.7 cells did not significantly differ between vehicle- and nalmefene-treated groups (data not shown). However, the mechanism by which nalmefene specifically increases CD36 expression, without affecting LOX-1 and SR-A, remains unclear. Further experiments are necessary to elucidate this mechanism.

Additionally, opioids and opioid receptors are involved in peripheral inflammation, the immune system, and phagocytosis, playing crucial roles in atherosclerotic plaque formation [11]. Nalmefene decreased the mRNA levels of mu-, delta-, and kappa-opioid receptors in RAW264.7 cells, which may promote inflammation and phagocytosis by reducing the expression of opioid receptors. Therefore, nalmefene may enhance the inflammatory response and phagocytosis by altering the expression of opioid receptors, leading to atherosclerotic plaque formation. However, the expression of interleukin-1β and Rela (p65) did not differ between vehicle- and nalmefene-treated RAW264.7 cells (data not shown). On the other hand, it may be involved in changes in the expression of other inflammatory cytokines such as tumor necrosis factor (TNF)-α, because each selective antagonist of mu-, delta-, and kappa-opioid receptors suppress the neuroinflammatory response by decreasing TNF-α expression in mouse microglia cell line [19]. Little is known about the mechanism of nalmefene-induced inflammation. Further experiments are needed to investigate this mechanism. In this study, we examined the in vitro effects of nalmefene on macrophages. However, opioid receptors are expressed in various cells of animals and humans, such as vascular smooth muscle cells, endothelial cells, and T cells [[20], [21], [22], [23], [24], [25]]. Kappa-opioid receptor stimulation alleviates vascular smooth muscle cell calcification [26] and improves endothelial function in hyperlipidemic rats [21,27]. Therefore, nalmefene may exert various effects on vascular smooth muscle cells, endothelial cells, and T cells, all of which play important roles in atherosclerosis. However, little is known about the effects of nalmefene in these cells. Further studies are required to clarify the mechanism of nalmefene-induced atherosclerotic plaque formation in macrophages and other cells in vitro.

In conclusion, nalmefene may promote the formation and vulnerability of atherosclerotic plaques by elevating plasma cholesterol levels. Furthermore, the drug may directly boost oxLDL uptake in macrophages by upregulating the expression of CD36. Additionally, it might induce pro-inflammation and phagocytosis by downregulating the expression of opioid receptors in macrophages. Consequently, caution is advised when using nalmefene in patients at risk of cardiovascular events.

## CRediT authorship contribution statement

**Mitsuhisa Koga:** Writing – original draft, Investigation, Funding acquisition, Formal analysis, Data curation, Conceptualization. **Koshun Inada:** Investigation, Data curation. **Ayano Yamada:** Investigation. **Kana Maruoka:** Investigation. **Atsushi Yamauchi:** Methodology.

## Declaration of competing interest

The authors declare that they have no known competing financial interests or personal relationships that could have appeared to influence the work reported in this paper.
